# The Efficacy of an mHealth App in Facilitating Weight Loss Among Japanese Fitness Center Members: Regression Analysis Study

**DOI:** 10.2196/48435

**Published:** 2023-11-08

**Authors:** Akifumi Eguchi, Yumi Kawamura, Takayuki Kawashima, Cyrus Ghaznavi, Keiko Ishimura, Shun Kohsaka, Satoru Matsuo, Shinichiro Mizuno, Yuki Sasaki, Arata Takahashi, Yuta Tanoue, Daisuke Yoneoka, Hiroaki Miyata, Shuhei Nomura

**Affiliations:** 1 Center for Preventive Medical Sciences Chiba University Chiba Japan; 2 Department of Health Policy and Management School of Medicine Keio University Tokyo Japan; 3 Department of Mathematical and Computing Science Tokyo Institute of Technology Tokyo Japan; 4 Department of Medicine University of California San Francisco San Francisco, CA United States; 5 Link & Communication Inc Tokyo Japan; 6 Department of Cardiology School of Medicine Keio University Tokyo Japan; 7 Communication Design Division RENAISSANCE INC Tokyo Japan; 8 Faculty of Marine Technology Tokyo University of Marine Science and Technology Tokyo Japan; 9 Center for Surveillance, Immunization, and Epidemiologic Research National Institute of Infectious Diseases Tokyo Japan; 10 Tokyo Foundation for Policy Research Tokyo Japan; 11 Department of Global Health Policy Graduate School of Medicine The University of Tokyo Tokyo Japan

**Keywords:** digital health, gym attendance, Japan, real-world data, smartphone application, weight loss

## Abstract

**Background:**

Self-tracking smartphone apps have emerged as promising tools to encourage healthy behaviors. In this longitudinal study, we used gym use data from members of a major fitness club that operates gyms throughout Japan from January 2014 to December 2019.

**Objective:**

Our objective was to assess the extent to which a health and fitness self-tracking mobile app introduced to gym members on January 1, 2018, contributed to their weight loss. The app allows users to input information regarding diet, sleep, weight, and gym exercise so that they can receive personalized feedback from an artificial intelligence chatbot to improve their health behaviors.

**Methods:**

We used linear regression to quantify the association between app use and weight loss. The primary outcome of the study was the weight loss achieved by each gym user, which was calculated as the difference between their initial and final weights in kilograms, as recorded in the app. Individuals who did not attend the gym or failed to use the mobile app at least twice during the study period were excluded from the analysis. The model accounted for age, gender, distance between the gym and the member’s residence, average weekly number of times a member used the gym, user’s gym membership length in weeks, average weekly number of times a member input information into the app, and the number of weeks that the app was used at least once.

**Results:**

Data from 26,589 participants were analyzed. Statistically significant associations were detected between weight loss and 2 metrics related to app use: the average weekly frequency of use and the total number of weeks in which the app was used at least once. One input per week was found to be associated with a loss of 62.1 (95% CI 53.8-70.5) g, and 1 week of app use was associated with 21.7 (95% CI 20.5-22.9) g of weight loss from the day of the first input to that of the final input to the app. Furthermore, the average number of times that a member used the gym weekly was also shown to be statistically significantly associated with weight loss: 1 use per week was associated with 255.5 (95% CI 228.5-282.6) g of weight loss.

**Conclusions:**

This empirical study demonstrated a significant association between weight loss among gym members and not only the frequency of weekly gym use but also the use of a health and fitness self-tracking app. However, further work is needed to examine the mechanisms through which mobile apps affect health behaviors and to identify the specific app features that are most effective in promoting weight loss.

## Introduction

In today’s digital era, the ubiquitous nature of smartphones and their apps has transformed health and fitness management. Self-tracking using smartphone apps has not only offered insights into one’s health but also reinforced behaviors that promote well-being [1,2]. As per the latest statistics, smartphones have become an integral health management tool. For example, on the App Store (Apple Inc) as of 2022, health and fitness ranked 8th out of 20 categories, accounting for 5.08% of all available apps [3]; on Google Play (Google LLC), health and fitness apps ranked 12th out of 33, accounting for 3.87% of all available apps [4].

Mobile health (mHealth) apps have the potential to overcome several challenges, such as the cost, burden, and adherence typical of traditional face-to-face health and fitness behavior promotion programs (eg, smoking and obesity control) [2,5,6]. They contain features that help people proactively manage their health, such as encouraging goal setting for healthy eating and exercise, self-monitoring of energy and nutrient intake, and weight tracking in the general population, some disease-affected groups, or populations at risk for certain lifestyle-related diseases [7]. People carry their smartphones for a significant amount of time during the day and provide numerous opportunities for real-time interventions throughout the day, whereas face-to-face health and fitness behavior promotion programs allow for contact only a few times a week.

On the other hand, the effectiveness of mobile apps on health-related behavioral changes and health outcomes has not yet reached a definitive conclusion. Indeed, Iribarren et al [8] and Milne-Ives et al [9] both conducted systematic reviews on the effectiveness of mHealth apps in influencing health outcomes. Iribarren et al [8] concluded that apps generally have a weak yet positive effect on health outcomes. On the other hand, Milne-Ives et al [9] observed that despite apps having high engagement and user satisfaction, the evidence supporting their ability to drive behavioral change is limited. To advance the field, there is a necessity for more rigorous, high-quality evidence before mHealth interventions are widely adopted [10]. Further studies among various segments of the population with different health conditions are needed to establish the effectiveness of mobile apps [11].

In light of the above, this study collaborates with a major Japanese fitness club that operates more than 100 gyms throughout the country. They have recently introduced an artificial intelligence (AI) advice app to help gym users manage their health and fitness. We hypothesized that there exists an association between the use of a gym-associated app specifically designed to support gym attendance and activities and weight loss among gym users. Validating this hypothesis is important; if a tangible relationship between supportive app engagement and weight loss is identified, it would reinforce recent evidence of the efficacy of integrating digital tools with traditional fitness approaches [12-15]. Such revelations could guide the design and enhancement of future digital health tools, ensuring they effectively bolster gym users’ weight management and fitness goals. Therefore, for this study, we obtained gym user attendance and app use data and used a linear regression method in order to assess the contribution of app use to weight loss.

## Methods

### App Details

The smartphone app referenced in this study is Calomama Plus (Link & Communication Inc), an AI advice app developed for gym members of RENAISSANCE INC. App users voluntarily register (self-report) information about their daily diet, sleep, weight, and gym exercise, and the app calculates caloric intake and expenditure [16]. The AI, supervised by experts, responds to such data and provides personalized feedback tailored to the user’s needs (such as maintaining health, improving metabolic syndrome, counteracting malnutrition, and preventing dementia), aiming to improve their health and fitness behaviors.

User registration for the app is simple; users agree to the terms of service and privacy policies and create a profile. Through the input of the RENAISSANCE gym membership identifier, integration with gym records is completed. Calomama Plus is compatible with various wearable tech devices, streamlining the data entry process through auto-acquisition. The app also incentivizes continued adherence through a reward-based point system. Detailed information about the interface and other features of this app, complete with videos, is available elsewhere [17].

### Data Used for Analysis

From RENAISSANCE, we sourced data from January 17, 2014, to December 31, 2019, concerning all gym members who had consented to third-party data sharing for research purposes at the time of membership. The data include each user’s number of gym visits (based on gym attendance records), gym membership length (based on the membership start date), and the distance from the gym to the user’s home (calculated by RENAISSANCE from the gym and home addresses), as well as age and gender at the time of membership enrollment. In relation to the app, data were obtained from Link & Communication on the number of inputs made by gym members using the app during their gym membership period. Any type of input (eg, weight, meals, sleep) is counted as 1 input. These data solely represent the frequency and intensity of app use, and no restrictions have been placed on the type of information. Additionally, the target weight set during the initial gym visit and self-reported height and weight records were also obtained.

### Study Population and Exclusion Criteria

To ascertain the influence of the app on gym users’ weight loss, this study focuses on adults aged between 20 and 80 years, who are considered to be less influenced by third parties, such as family members, regarding their own health and fitness behaviors. Gym users with target weights of less than 30 kg or greater than 150 kg, those with a BMI of less than 16 or greater than 40, or those with weight changes of greater than 50 kg were excluded from this analysis.

RENAISSANCE began introducing and recommending the use of the Calomama Plus app to support gym users in managing their health and fitness on January 1, 2018, when it became available for download on the App Store and Google Play. Data from 2 gyms that began app introduction on a trial basis on October 1, 2017, were excluded from this analysis.

The data period for this analysis was from January 1, 2018, to December 31, 2019, during which time the app was available. Individuals who either did not attend the gym or used the app fewer than twice during the study period were excluded from the analysis. Individuals who had not entered the necessary data into the app were also excluded from the analysis.

There are two main patterns in which users use gyms: (1) they freely use the facilities for health and fitness purposes on their own schedule, or (2) they participate in yoga and sports training sessions offered by the gym on a fixed schedule. The gym attendance data contained unique identifiers for each pattern. Since the latter pattern of gym attendance is greatly influenced by the management policies of individual gyms, only the former is considered in this study.

### Primary Analysis

The outcome of this study was weight loss, defined as the difference between the first and final input weights in the app.

Upon commencing their gym routines, participants recorded their primary fitness objectives—either weight loss or weight gain—within the app. Out of the total 28,840 individuals pooled during the study duration, a substantial 92.2% (n=26,589 individuals) targeted weight loss, while a mere 7.8% (n=2251 individuals) aimed at weight gain. Due to this pronounced difference and to uphold statistical robustness, especially when considering stratified analyses based on gym attendance frequency (see below), this study exclusively focused on those targeting weight loss. Moreover, gym users tend to establish clear goals based on body weight, which is more intuitively graspable than BMI. Given these practical considerations, this study uses body weight as its primary outcome measure instead of BMI.

In this study, we used a linear regression method. To adjust for confounding, the model accounted for age, gender, distance between the gym and the member’s residence, average weekly number of times a member used the gym, user’s gym membership length in weeks, average weekly number of times a member input information into the app, and the number of weeks that the app was used at least once. The distance between the gym and the attendee’s place of residence was calculated and categorized by RENAISSANCE (0-0.5 km, 0.5-1 km, 1-2 km, 2-3 km, 3-4 km, 4-5 km, 5-6 km, 6-7 km, 7-8 km, 8-9 km, and more than 9 km). To preserve anonymity, we only received categorized data as opposed to the location of attendees’ residences.

### Sensitivity Analysis

We validated the results by performing the same regression analysis after stratifying users into 3 groups based on 1 of 2 sets of criteria for the frequency of gym attendance between January 17, 2014, and December 31, 2019 (average monthly number of times a person used the gym: 0-4 times, 4-8 times, and more than 8 times; 0-3 times, 3-7 times, and more than 7 times).

### Ethical Considerations

Ethical approval was granted by the Research Ethics Committee of Keio University School of Medicine (202000566). All the obtained data were anonymized, and confidentiality was maintained. No compensation or remuneration was given to the research participants. Informed consent was obtained from all individuals involved in the study.

## Results

### Demographic Characteristics

Demographic characteristics of the sample—age, gender, distance from home to the gym, average weekly number of times a person used the gym, user’s gym membership length in weeks, average weekly number of times a member input information into the app, and the number of weeks that the app was used at least once—both stratified by frequency of gym attendance and in total are displayed in Table 1. In total, we analyzed 26,589 gym members. The average age was 38.9 (SD 12.9) years, and 63.8% (16,976/26,589) identified as male. The average body weight of participants initially inputted into the app was 64.3 (SD 13.2) kg, and the corresponding average BMI was 24.0 (SD 3.7) kg/m^2^. Approximately 33.2% (8814/26,589) of members lived within 1 km of their respective gym. On average, members used their gym approximately 1.4 times (SD 1.0) per week. The average length of gym membership was 61.2 (SD 70.9) weeks, and the average number of weeks that the app was used at least once was 19.7 (SD 25.2). The average weekly number of times a member inputted information into the app was 4.7 (SD 3.4).

**Table 1:** Demographic characteristics of gym users sampled from January 17, 2014, to December 31, 2019.

Demographic		Overall (n=26,589)	Stratification by frequency of gym attendance (average monthly number of times a person used the gym)					
			0-4 times (n=12,075)	4-8 times (n=8984)	More than 8 times (n=5530)	0-3 times (n=8028)	3-7 times (n=11,447)	More than 7 times (n=7114)
Age (years), mean (SD)		38.91 (12.86)	37.17 (12.13)	38.88 (12.71)	42.77 (13.81)	36.58 (11.89)	38.46 (12.55)	42.28 (13.70)
**Gender, n (%)**								
	Female	9613 (36.2)	4030 (33.4)	3249 (36.2)	2334 (42.2)	2664 (33.2)	4051 (35.4)	2898 (40.7)
	Male	16,976 (63.8)	8045 (66.6)	5735 (63.8)	3196 (57.8)	5364 (66.8)	7396 (64.6)	4216 (59.3)
Weight (kg), mean (SD)		64.3 (13.2)	63.7 (13.1)	64.4 (13.1)	65.2 (13.3)	63.9 (13.2)	64.1 (13.1)	65.0 (13.3)
Height (cm), mean (SD)		163.1 (8.4)	162.7 (8.4)	163.2 (8.3)	163.6 (8.5)	162.7 (8.3)	163.0 (8.4)	163.5 (8.5)
BMI (kg/m^2^), mean (SD)		24.0 (3.7)	23.9 (3.7)	24.0 (3.6)	24.2 (3.7)	24.0 (3.7)	24.0 (3.6)	24.1 (3.7)
**Distance from gym (km), n (%)**								
	0-0.5	3481 (13.1)	1548 (12.8)	1135 (12.6)	798 (14.4)	1074 (13.4)	1397 (12.2)	1010 (14.2)
	0.5-1	5333 (20.1)	2440 (20.2)	1773 (19.7)	1120 (20.3)	1647 (20.5)	2256 (19.7)	1430 (20.1)
	1-2	6768 (25.5)	3054 (25.3)	2288 (25.5)	1426 (25.8)	2053 (25.6)	2906 (25.4)	1809 (25.4)
	2-3	3906 (14.7)	1779 (14.7)	1352 (15.0)	775 (14.0)	1144 (14.3)	1746 (15.3)	1016 (14.3)
	3-4	2401 (9.0)	1125 (9.3)	787 (8.8)	489 (8.8)	738 (9.2)	1025 (9.0)	638 (9.0)
	4-5	1716 (6.5)	779 (6.5)	578 (6.4)	359 (6.5)	505 (6.3)	754 (6.6)	457 (6.4)
	5-6	1039 (3.9)	473 (3.9)	383 (4.3)	183 (3.3)	301 (3.7)	493 (4.3)	245 (3.4)
	6-7	701 (2.6)	321 (2.7)	245 (2.7)	135 (2.4)	203 (2.5)	313 (2.7)	185 (2.6)
	7-8	530 (2.0)	222 (1.8)	200 (2.2)	108 (2.0)	151 (1.9)	237 (2.1)	142 (2.0)
	8-9	383 (1.4)	167 (1.4)	136 (1.5)	80 (1.4)	100 (1.2)	178 (1.6)	105 (1.5)
	>9	331 (1.2)	167 (1.4)	107 (1.2)	57 (1.0)	112 (1.4)	142 (1.2)	77 (1.1)
Average weekly number of times a member used the gym, mean (SD)		1.38 (0.99)	0.61 (0.24)	1.44 (0.28)	2.94 (0.86)	0.48 (0.18)	1.18 (0.28)	2.71 (0.88)
User’s gym membership length (weeks), mean (SD)		61.19 (70.94)	56.85 (61.58)	58.88 (70.42)	74.41 (87.38)	58.68 (62.65)	56.12 (65.64)	72.18 (85.30)
Average weekly number of times a member input information into the app, mean (SD)		4.71 (3.36)	4.55 (3.41)	4.76 (3.32)	4.98 (3.29)	4.53 (3.44)	4.71 (3.35)	4.92 (3.28)
The number of weeks that the app was used at least once, mean (SD)		19.68 (25.24)	18.91 (24.22)	19.33 (24.81)	21.95 (27.86)	19.21 (24.41)	18.90 (24.45)	21.49 (27.26)

### Linear Regression Analysis

The results of the linear regression analysis are shown in Table 2. Age was not found to significantly predict weight loss, but male gender was significantly associated with 280.9 (95% CI 225.5-336.2) g of weight loss compared to women. The average weekly number of times that a member used the gym was significantly associated with weight loss: each additional use was associated with 255.5 (95% CI 228.5-282.6) g of weight loss. The length of the user’s gym membership was associated with a slight but significant weight gain of 2.6 (95% CI 2.2-3.0) g. The average weekly number of times that a member input information into the app and the number of weeks that the app was used at least once were also significantly associated with weight loss: each additional input and week were associated with 62.1 (95% CI 53.8-70.5) g and 21.7 (95% CI 20.5-22.9) g of weight loss.

The results of the sensitivity analyses, which were conducted using groups stratified by the frequency of gym attendance, are shown in Tables 3 and 4. Specifically, Table 3 uses stratification groups of 1-4, 4-8, and ≥8 times per month, whereas Table 4 is based on groups of 1-3, 3-7, and ≥7 times per month. Age remained a poor predictor of weight change, while the male gender continued to be associated with significant weight loss compared to women in all groups. The average weekly number of times that a member used the gym was significantly associated with weight loss in 5 out of 6 groups. The length of the user’s gym membership continued to predict a slight but significant weight gain in 4 of 6 groups. The average weekly number of times that a member inputs information into the app remained a significant predictor of weight loss in all groups, and the effect size increased monotonically with gym use frequency; the same pattern was observed with the number of weeks that the app was used at least once.

**Table 2:** Linear regression of demographic characteristics, gym use, and app use versus weight change.

Covariates and characteristics		Estimates (95% CI)	*P* value
Age (years)		1.31 (–0.90 to 3.52)	.25
**Gender**			
	Female	–280.85 (–336.18 to –225.53)	.001
	Male	Reference	N/A^a^
**Distance between the gym and the member’s residence (km)**			
	0-0.5	20.01 (–73.57 to 113.58)	.68
	0.5-1	Reference	N/A
	1-2	53.28 (–25.39 to 131.95)	.18
	2-3	71.62 (–18.89 to 162.13)	.12
	3-4	86.85 (–18.73 to 192.42)	.11
	4-5	49.7 (–69.50 to 168.91)	.41
	5-6	97.46 (–48.17 to 243.09)	.19
	6-7	–59.1 (–231.61 to 113.41)	.50
	7-8	–5.17 (–200.73 to 190.38)	.96
	8-9	13.83 (–213.32 to 240.97)	.91
	>9	526.53 (283.27 to 769.80)	.001
Average weekly number of times a member used the gym		255.52 (228.48 to 282.56)	.001
User’s gym membership length in weeks		–2.56 (–2.97 to –2.15)	.001
Average weekly number of times a member inputted information into the app		62.13 (53.81 to 70.46)	.001
The number of weeks that the app was used at least once		21.71 (20.54 to 22.88)	.001

^a^N/A: not applicable.

**Table 3:** Linear regression analysis stratified by gym use frequency: groups of 1-4, 4-8, and ≥8 times per month.

Analysis		Frequency of gym attendance (average monthly number of times a person used the gym)					
		0-4 times, estimates (95% CI)	*P* value	4-8 times, estimates (95% CI)	*P* value	More than 8 times, estimates (95% CI)	*P* value
Age (years)		2.60 (–0.24 to 5.44)	.07	1.32 (–2.37 to 5.00)	.48	0.14 (–5.83 to 6.11)	.96
**Gender**							
	Female	–146.65 (–217.07 to –76.23)	.001	–303.9 (–395.55 to –212.24)	.001	–474.37 (–627.77 to –320.96)	.001
	Male	Reference	N/A^a^	Reference	N/A	Reference	N/A
**Distance between the gym and the member’s residence (km)**							
	0-0.5	21.24 (–95.92 to 138.40)	.72	42.84 (–14.92 to 200.59)	.59	–7.08 (–266.73 to 2.57)	.96
	0.5-1	Reference	N/A	Reference	N/A	Reference	N/A
	1-2	39.12 (–58.87 to 137.12)	.43	26.43 (–104.93 to 157.79)	.69	110.90 (–112.97 to 334.77)	.33
	2-3	81.77 (–30.78 to 194.32)	.15	30.72 (–119.26 to 180.70)	.69	95.88 (–166.16 to 357.93)	.47
	3-4	64.50 (–65.58 to 194.57)	.33	110.24 (–67.62 to 288.10)	.22	26.81 (–277.14 to 330.76)	.86
	4-5	6.93 (–141.50 to 155.36)	.93	69.13 (–129.71 to 267.97)	.50	54.72 (–285.55 to 394.99)	.75
	5-6	129.37 (–51.83 to 310.57)	.16	73.90 (–159.99 to 307.79)	.54	36.17 (–410.97 to 483.31)	.87
	6-7	15.17 (–198.97 to 229.3)	.89	–37.72 (–320.53 to 245.09)	.79	–307.01 (–817.7 to 203.68)	.24
	7-8	–224.22 (–477.08 to 28.65)	.08	–13.49 (–322.96 to 295.99)	.93	338.82 (–226.05 to 903.69)	.24
	8-9	80.02 (–208.42 to 368.46)	.59	–78.24 (–447.41 to 290.93)	.68	1.02 (–647.56 to 649.61)	.996
	>9	174.03 (–114.5 to 462.56)	.24	469.07 (55.96 to 882.18)	.03	1481.38 (720.02 to 2242.74)	.001
Average weekly number of times a member used the gym		222.42 (85.25 to 359.60)	.001	149.97 (–4.23 to 304.17)	.06	137.69 (49.6 to 225.78)	.002
User’s gym membership length in weeks		–0.43 (–1.00 to 0.15)	.15	–2.51 (–3.20 to –1.82)	.001	–5.29 (–6.27 to –4.31)	.001
Average weekly number of times a member inputted information into the app		41.12 (30.75 to 51.50)	.001	50.65 (36.74 to 64.56)	.001	108.29 (84.35 to 132.23)	.001
The number of weeks that the app was used at least once		13.48 (11.95 to 15.01)	.001	21.07 (19.09 to 23.05)	.001	35.46 (32.46 to 38.47)	.001

^a^N/A: not applicable.

**Table 4:** Linear regression analysis stratified by gym use frequency of 1-3, 3-7, and ≥7 times per month.

Analysis			Frequency of gym attendance (average monthly number of times a person used the gym)										
			0-3 times, estimates (95% CI)		*P* value		3-7 times, estimates (95% CI)		*P* value		More than 7 times, estimates (95% CI)		*P* value
Age (year)			1.05 (–2.51 to 4.61)		.56		3.27 (0.14 to 6.39)		.04		–0.41 (–5.44 to 4.62)		.87
**Gender**													
	Female	–156.21 (–242.85 to -69.57)		.001		–262.14 (–340.42 to –183.86)		.001		–414.72 (–543.81 to –285.63)		.001	
	Male	Reference		N/A^a^		Reference		N/A		Reference		N/A	
**Distance between the gym and the member’s residence (km)**													
	0-0.5	78.53 (–63.07 to 220.14)		.28		–8.02 (–143.23 to 127.18)		.91		7.25 (–211.36 to 225.86)		.95	
	0.5-1	Reference		N/A		Reference		N/A		Reference		N/A	
	1-2	57.12 (–62.46 to 176.70)		.35		13.75 (–97.76 to 125.27)		.81		92.15 (–96.10 to 280.40)		.34	
	2-3	117.37 (–21.78 to 256.53)		.10		10.69 (–116.00 to 137.38)		.87		98.53 (–119.86 to 316.92)		.34	
	3-4	44.65 (–115.50 to 204.80)		.58		88.35 (–61.33 to 238.04)		.25		73.23 (–180.08 to 326.54)		.57	
	4-5	41.44 (–142.33 to 225.21)		.69		24.29 (–142.80 to 191.38)		.78		54.35 (–231.73 to 340.44)		.71	
	5-6	192.18 (–34.31 to 418.66)		.10		–59.81 (–257.24 to 137.62)		.55		253.67 (–114.35 to 621.69)		.18	
	6-7	17.12 (–251.56 to 285.81)		.90		–29.99 (–269.49 to 209.51)		.81		–241.31 (–656.83 to 174.20)		.26	
	7-8	–329.23 (–636.42 to -22.05)		.04		45.85 (–225.36 to 317.05)		.74		195.36 (–272.63 to 663.35)		.41	
	8-9	–35.53 (–407.38 to 336.33)		.85		11.91 (–297.24 to 321.06)		.94		22.21 (–515.48 to 559.90)		.94	
	>9	208.94 (–143.72 to 561.61)		.25		218.96 (–124.63 to 562.56)		.21		1493.45 (871.04 to 2115.87)		.001	
Average weekly number of times a member used the gym			243.5 (15.51 to 471.50)		.04		328.17 (197.49 to 458.85)		.001		196.58 (124.27 to 268.89)		.001
User’s gym membership length in weeks			–0.03 (–0.72 to 0.67)		.94		–2.23 (–2.86 to –1.61)		.001		–4.61 (–5.45 to –3.77)		.001
Average weekly number of times a member inputted information into the application			38.81 (26.11 to 51.51)		.001		49.57 (37.83 to 61.31)		.001		97.69 (77.52 to 117.85)		.001
The number of weeks that the application was used at least once			11.63 (9.76 to 13.50)		.001		20.86 (19.16 to 22.57)		.001		31.52 (28.96 to 34.09)		.001

^a^N/A: not applicable.

## Discussion

### Overview

Using gym registration data from a major Japanese sports club that operates more than 100 gyms throughout the country, we assessed whether the use of an AI health and fitness app was associated with weight loss among gym users. There were statistically significant associations between weight loss and 2 metrics related to app use: the average weekly frequency of use and the total number of weeks in which the app was used at least once. Furthermore, we found that app use predicted more weight loss among those who used the gym more often.

These findings suggest that health apps can play a crucial role in promoting weight loss among gym users, acting as a complementary tool that enhances the effects of physical exercise. These apps may facilitate adherence to exercise routines, offer consistent monitoring, and supplement gym activities with valuable dietary and lifestyle advice [18,19]. Moreover, it was observed that increased gym use itself is directly linked to greater weight loss, indicating that the combination of physical exercise and digital guidance creates a holistic approach to managing weight.

The use of smartphone apps to encourage healthy behaviors has been the subject of significant attention, and several systematic reviews and meta-analyses have attempted to determine their efficacy. According to meta-analyses performed by Mateo et al [14] and Islam et al [15], the use of mHealth apps was associated with an overall decrease in body weight and BMI of approximately 1 kg and 0.5 kg/m^2^, respectively. Furthermore, a review of randomized control trials specifically focusing on Asian populations found a small-to-moderate effect size for weight loss among those in the mHealth app group [20]. mHealth app–associated weight loss has been found to be sustained for up to 12 months, though weight loss was most robust earlier in the intervention, around 3 months [21]. The findings of this study are largely consistent with those of the previous literature, which suggests that app use is associated with weight loss, though the degree of the effect size is variable.

The Calomama Plus app allows users to input information regarding their diet, sleep, weight, and gym attendance in order to calculate caloric intake and expenditure. Using this data, the app then provides personalized feedback to the users to promote healthy behaviors such as an improved diet and increased exercise, which may manifest in weight loss. By encouraging self-monitoring of diet and health behaviors, the app encourages adherence to healthy behaviors [22]. Notably, the fact that phones are portable and accessible around the clock confers significant advantages to mHealth interventions compared to traditional coaching methods [14]. Evidence suggests that greater engagement with smartphone apps may translate into more robust outcomes [13,23]. Our results were consistent with past findings in that those who used the app more often were predicted to have higher degrees of weight loss. Notably, engagement with physical activity apps and the beneficial effects of engagement may wane over time [24], so efforts are needed to enhance the user experience such that app users continue to engage with mobile interventions in the long term. Personalized feedback has been described as one of the primary advantages of mHealth interventions [15]; the AI chatbot that provides personalized feedback to users likely played a role in the success of Calomama Plus in promoting weight loss.

The effects of mHealth apps on physical activity, specifically, have yielded mixed results. According to a review conducted by Emberson et al [25], of 19 articles assessing the effects of smartphone-based interventions on health, 11 demonstrated a positive effect on at least one of the following: moderate to vigorous physical activity, step count, sedentary behavior, cardiorespiratory fitness, and blood pressure. Similarly, a meta-analysis of randomized controlled trials for smartphone app effects on physical activity found a statistically significant increase of 1850 steps per day [26]. Contrastingly, a similar meta-analysis found a nonsignificant increase of 477 steps per day [24]. Furthermore, a systemic review of 52 randomized controlled trials by Milne-Ives et al [9] found no significant evidence that mHealth apps changed behaviors or health outcomes. Though this study did not specifically test whether the use of the app changed gym attendance, we found that app use predicted higher weight loss among those who had higher gym attendance.

### Strengths and Limitations

The strengths of this study include a large sample size (n=26,589) aligned with real-world data, making the present analysis one of the largest empirical studies of the effects of smartphone app use on weight loss to date. Furthermore, this study considers a 2-year period and allows for a long-term assessment of the effects of the smartphone app, as the process of losing weight often occurs over months or years. Finally, gym attendance was monitored directly by the sports club, thus avoiding the potential effects of reporting bias that are often present in studies assessing the relationship between physical health behaviors and health outcomes.

However, this study also has some notable limitations. Foremost among these is our inability to compare the weight data between the users of the app and nonusers. The weight data is linked solely to the app users, rendering it impossible to establish a comparison group with those who did not use the app. Consequently, we cannot infer a causal relationship pertaining to the app’s efficacy from this data set alone. Second, although the app was developed for gym users managed by RENAISSANCE and its use was actively encouraged, not all users installed the app. Third, if there were confounding events that could have increased or decreased gym use during this 2-year period, we would not have been able to account for them in this analysis. Fourth, weight and BMI alone are not all-encompassing indicators of health, and we did not have access to the body compositional data of the participants in this study [27], which may have shed further light on the state of their physical wellness. Studies that consider the effects of mHealth apps on body composition represent an interesting avenue for future research. Fifth, self-reported body weight in the study can be limited by potential reporting bias, which can introduce errors and skew conclusions drawn from the data [28]. However, in studies like ours, where the primary motivation reported by participants is personal health and weight management, discrepancies between self-reported and actual weight might be minimized. Additionally, considering the scope of this study in a gym environment where scales are readily available, that discrepancy is likely to be small. Finally, this limited scope of this study, focused on a single app and restricted to data from a single gym company, greatly affects the generalizability of the research results.

### Conclusions

We found that, in addition to the frequency of weekly gym use, the frequency and continuity with which a health and fitness self-tracking app was used were associated with a decrease in weight among a sample of over 20,000 gym members in Japan. Future work is needed to examine the mechanisms by which apps influence health behaviors and what app features are effective for sustaining wellness-promoting health behaviors.

## Abbreviations

AI: artificial intelligence
mHealth: mobile health

## Data Availability

The data sets generated during and/or analyzed during this study are not publicly available due to ethical considerations but are available from the corresponding author on reasonable request.
